# Cognitive Impairment and Dysphagia Associated with Postoperative Hydrocephalus Secondary to Cerebrospinal Fluid Leakage After C1–2 Fusion: A Case Report

**DOI:** 10.3390/jcm15145470

**Published:** 2026-07-13

**Authors:** Pureum Kim, Jung Jae Lee, Dongwon Lee, Sook Joung Lee

**Affiliations:** 1 Department of Physical Medicine and Rehabilitation, Daejeon St. Mary’s Hospital, College of Medicine, The Catholic University of Korea, Seoul 06591, Republic of Korea; vnfma6163@naver.com (P.K.); iwoner97@naver.com (D.L.); 2 Department of Neurosurgery, Daejeon St. Mary’s Hospital, College of Medicine, The Catholic University of Korea, Seoul 06591, Republic of Korea

**Keywords:** cognitive impairment, dysphagia, giant pseudomeningocele, hydrocephalus, upper cervical fusion, personalized rehabilitation

## Abstract

**Introduction:** Posterior cervical fusion is commonly performed for cervical spine instability and fractures. However, dural injury may result in cerebrospinal fluid leakage, pseudomeningocele, and, rarely, hydrocephalus. These complications may lead to substantial neurological deterioration and functional decline. We report a rare case of severe cognitive impairment and dysphagia caused by postoperative CSF leakage-associated hydrocephalus after C1–2 fusion and highlight the role of comprehensive rehabilitation in functional recovery. **Case presentation**: A 67-year-old woman sustained a type III odontoid fracture and was initially managed with halo-vest immobilization. Because of fracture nonunion, posterior C1–2 fusion was performed. One month after surgery, she developed progressive mental status deterioration, confusion, and severe functional impairment. Brain computed tomography revealed progressive hydrocephalus, and cervical spine magnetic resonance imaging demonstrated a large posterior fluid collection consistent with pseudomeningocele. Revision surgery and ventriculoperitoneal shunt placement were performed. However, the patient remained bedridden with persistent vomiting, severe cognitive impairment, dysphagia requiring nasogastric tube feeding, and generalized deconditioning. Three months after revision surgery, her neurological condition stabilized and rehabilitation was initiated. Intensive and individualized rehabilitation, including cognitive rehabilitation, swallowing therapy was implemented. After three months of rehabilitation, the patient demonstrated gradual improvement in cognitive function, swallowing function, and mobility, ultimately achieving independent standing and ambulation using a walker. **Conclusions**: Postoperative CSF leakage-associated hydrocephalus and pseudomeningocele may lead to severe cognitive, swallowing, and functional impairments beyond structural complications alone. Comprehensive, individualized rehabilitation should be considered an essential component of care to maximize neurological and functional recovery.

## 1. Introduction

C1–C2 fusion is a commonly performed surgical procedure for the treatment of upper cervical instability. The upper cervical region contains critical anatomical structures, including the dura mater, spinal cord, and vertebral vessels. Because surgical procedures are performed in close proximity to these structures, various postoperative complications may occur [1].

Dural tears have an estimated incidence of 0.8% to 3.9% following posterior cervical spine surgery, with 7% to 32% of these cases requiring additional intervention because of persistent cerebrospinal fluid (CSF) leakage [2]. Pseudomeningocele (PMC) following spinal surgery is a condition in which CSF continuously leaks through a compromised dura mater and accumulates within the surrounding soft tissue [3]. PMC is a relatively rare complication of cervical spine surgery and may resolve spontaneously over time [4]. However, persistent PMC may alter CSF dynamics and, in rare cases, lead to hydrocephalus [5]. These complications may result in significant neurological deterioration and profound functional decline.

To date, only a few cases of giant cervical pseudomeningocele have been reported in the literature [6,7]. Most previous reports have focused primarily on structural complications and their surgical management. However, cases in which postoperative CSF leakage progresses to PMC and subsequent hydrocephalus, leading to severe functional decline such as cognitive impairment and dysphagia, have rarely been described. Here, we report a patient who developed severe cognitive impairment and dysphagia secondary to postoperative CSF leakage-associated hydrocephalus following C1–2 fusion. This case highlights the potential role of comprehensive personalized rehabilitation in promoting meaningful recovery of cognitive and swallowing function.

## 2. Case Presentation

A 67-year-old woman presented with a type III odontoid fracture after a motor vehicle accident and initially underwent halo-vest immobilization for two months. However, after removal of the external fixation device, she continued to experience persistent tingling sensations in both hands. Follow-up imaging demonstrated fracture nonunion, and posterior C1–2 fusion was subsequently performed (Figure 1). One week after surgery, the patient developed headache. Brain computed tomography (CT) showed no remarkable abnormalities, and no definite postoperative complications were identified at that time. Therefore, she was discharged home.

One month after surgery, she presented to the emergency department with progressive neurological deterioration, including confusion, disorientation, severe memory impairment, and dysarthria. Brain CT demonstrated progressive hydrocephalus (Figure 2A,B), while cervical spine magnetic resonance imaging (MRI) demonstrated a large posterior fluid collection associated with CSF leakage, consistent with a giant PMC measuring approximately 91 × 63 × 40 mm (length × width × depth) (Figure 2C,D).

Accordingly, revision surgery for PMC and ventriculoperitoneal shunt placement for hydrocephalus were performed. Despite neurosurgical interventions, the patient’s functional condition did not substantially improve. She continued to experience persistent vomiting, cognitive dysfunction, and dysphagia, further aggravated by recurrent hypotensive episodes and severe generalized deconditioning. Consequently, she became unable to maintain independent standing or ambulation, and her functional status fluctuated between bedridden and wheelchair-dependent. Serial videofluoroscopic swallowing studies (VFSS) demonstrated persistent aspiration, accompanied by gastroesophageal reflux and nasopharyngeal regurgitation (Figure 3A,B). Therefore, nasogastric tube feeding was maintained. and rehabilitation interventions were restricted to supportive care and prevention of further deconditioning because of her unstable medical condition.

Three months after revision surgery, her neurological deterioration stabilized, and she was transferred to the Department of Rehabilitation Medicine. At that time, an active and comprehensive rehabilitation program was initiated. Initial rehabilitation assessment revealed severe cognitive impairment with marked disorientation, and the Mini-Mental State Examination (MMSE) score was 5. VFSS demonstrated persistent silent aspiration (Penetration–Aspiration Scale, PAS [7] score: 8). Although motor strength in all extremities was relatively preserved (fair-to-good grade), severe generalized deconditioning markedly limited her functional performance. As a result, she remained in a wheelchair-dependent state. Functional evaluation demonstrated profound disability, with a Modified Barthel Index (MBI) score of 11 and a Berg Balance Scale (BBS) score of 3 (Table 1).

An individualized and comprehensive rehabilitation program was initiated and progressively intensified according to the patient’s functional recovery. The patient participated in rehabilitation five days per week, receiving approximately 2–3 h of individualized therapy each day, including physical therapy, occupational therapy, cognitive rehabilitation, swallowing therapy and speech-language therapy.

Physical therapy focused on progressive mobilization. To improve orthostatic intolerance and facilitate participation in rehabilitation, tilt table training was initiated for orthostatic blood pressure stabilization. As hypotensive episodes gradually subsided, rehabilitation was advanced to sitting balance training, sit-to-stand training, and functional strengthening exercise, progressive gait training with a walker, and endurance training as tolerated.

Occupational therapy consisted of upper extremity training and robot-assisted hand rehabilitation. Cognitive rehabilitation included computer-based cognitive training and speech-language therapy. Swallowing rehabilitation consisted of neuromuscular electrical stimulation (VitalStim^®^ (Chattanooga, Hixson, TN, USA)), oral motor facilitation, pharyngeal muscle strengthening exercises, effortful swallowing, compensatory swallowing techniques (including postural modification and supraglottic swallowing), and dietary modification based on VFSS findings. Additional supportive interventions included pain-relieving modalities and pharmacologic management for symptom control, including analgesics, ginkgo extract, and proton pump inhibitors for reflux management.

After three months of comprehensive and intensive rehabilitation, the patient demonstrated gradual improvement in overall functional status, including cognitive function, swallowing function, and mobility. Cognitive function improved markedly, with the MMSE score increasing from 5 to 19. Disorientation was no longer observed, and the patient regained the ability to perform simple calculations and participate in daily meaningful communication. Furthermore, follow-up swallowing evaluation demonstrated complete resolution of aspiration (PAS score: 1), allowing advancement to a regular oral diet (Figure 3C). Ambulatory function improved substantially, and the patient regained the ability to stand independently and ambulate using a walker. Functional evaluation demonstrated substantial improvement, as reflected by an increase in the MBI from 11 to 67 and improvement in the BBS score from 3 to 39. Following significant neurological and functional recovery, the patient was transferred to a rehabilitation hospital for continued rehabilitation.

## 3. Discussion

Type III odontoid fractures can be managed either conservatively with halo-vest immobilization or surgically through stabilization procedures. However, because of the complex anatomy of the craniocervical junction, both treatment approaches carry distinct risks of complications. Halo-vest immobilization may result in pin-site infection, skull penetration, fracture nonunion, and aspiration pneumonia related to prolonged immobilization [8]. Posterior C1–C2 fusion may be associated with vertebral artery injury, nerve root injury, surgical site infection, and intraoperative dural tears leading to CSF leakage [1].

PMC following spine surgery is a rare complication, with a reported incidence of approximately 0.08% in a large multicenter study [4]. Weng et al. defined giant PMC as a lesion exceeding 8 cm in length [9] and reported that most cases occurred in the lumbar spine (82%), whereas cervical involvement was relatively uncommon (18%). Accordingly, only a limited number of cases of giant cervical PMC have been reported in the literature [6,9]. Hydrocephalus secondary to postoperative CSF leakage is even more uncommon and has primarily been reported as isolated case reports [5].

### 3.1. Pathophysiological Mechanisms of Postoperative Pseudomeningocele and Secondary Hydrocephalus

The pathophysiological mechanism underlying PMC formation is thought to begin with a dural defect, which permits CSF leakage into adjacent soft tissues. Initially, the leaked CSF may be partially reabsorbed [10]; however, persistent CSF extravasation and progressive connective tissue reaction can reduce local CSF resorption and lead to encapsulated fluid collection, resulting in PMC formation [6,9].

In rare cases, prolonged CSF leakage associated with a giant PMC may alter intracranial CSF homeostasis and contribute to secondary hydrocephalus [5]. Although the exact mechanism remains unclear, disruption of normal CSF circulation and pressure regulation has been proposed as a possible contributing factor. Furthermore, in our patient, progressive hydrocephalus was accompanied by severe cognitive impairment and dysphagia, suggesting that postoperative structural complications may progress beyond local anatomical abnormalities and lead to clinically significant functional deterioration.

### 3.2. Cognitive Dysfunction Associated with Postoperative Pseudomeningocele and Hydrocephalus: Implications for Rehabilitation

In our patient, postoperative secondary hydrocephalus likely contributed to severe cognitive impairment through disturbed CSF circulation and secondary disruption of cortical–subcortical networks involved in higher cognitive processing.

Although evidence is primarily derived from patients with idiopathic normal-pressure hydrocephalus (iNPH), previous studies provide supportive evidence that restoration of CSF circulation alone may not necessarily ensure complete cognitive recovery [11]. Koivisto et al. reported that among 146 shunt-responsive patients with idiopathic normal-pressure hydrocephalus (iNPH) followed for a median of 4.8 years, 80% developed cognitive decline and 46% eventually met criteria for dementia [12]. In a nationwide population-based study including 2053 patients with iNPH, Tseng et al. demonstrated that CSF shunt surgery was associated with a reduced long-term risk of Alzheimer disease; however, the overall risk of dementia remained incompletely eliminated [13]. Collectively, these studies suggest that restoration of CSF circulation alone may not necessarily ensure complete cognitive recovery in patients with iNPH. However, because postoperative secondary hydrocephalus and iNPH differ in their underlying etiologies and pathophysiological mechanisms, these findings should be interpreted cautiously in the context of our case.

Although our patient developed postoperative secondary hydrocephalus rather than iNPH, disturbed CSF circulation may also have contributed to the severe cognitive impairment observed in this case. Notably, substantial cognitive deficits persisted despite neurosurgical treatment but improved following individualized cognitive rehabilitation. Therefore, clinicians should consider serial cognitive assessment and rehabilitation strategies as part of long-term management for patients with hydrocephalus-associated cognitive dysfunction, even after successful structural treatment.

### 3.3. Dysphagia Following Upper Cervical Fusion: Multifactorial Mechanisms and Rehabilitation Implications

Dysphagia may also occur following posterior upper cervical fusion, including C1–2 fusion, although its reported incidence (9.5–26.3%) is lower than that of anterior cervical discectomy and fusion (ACDF), in which rates of up to 87.5% have been reported [14]. Unlike ACDF, where direct manipulation of anterior swallowing structures is considered a major mechanism, dysphagia after posterior C1–2 fusion is thought to be primarily related to postoperative changes in craniocervical alignment, which may reduce the oropharyngeal space and impair swallowing biomechanics [15].

However, dysphagia in our patient was unlikely to be explained by posterior C1–2 fusion alone. Progressive hydrocephalus may have contributed to impaired central swallowing control, while prolonged immobilization, recurrent vomiting, and severe generalized deconditioning likely compounded swallowing dysfunction. This multifactorial mechanism may explain the severity and persistence of dysphagia in our case and underscores the importance of comprehensive swallowing assessment and intensive dysphagia rehabilitation in addition to structural treatment.

Dysphagia accompanied by persistent vomiting may also place patients at substantial risk of malnutrition and dehydration during the postoperative period. Inadequate nutritional intake can further aggravate sarcopenia, delayed wound healing, and generalized deconditioning, all of which may adversely compromise neurological recovery and rehabilitation outcomes. Patients with prolonged dysphagia are also at risk of deficiencies in protein, energy, and micronutrients, including vitamin D, vitamin B12, folate, iron, and zinc, which may contribute to impaired muscle function, fatigue, and delayed functional recovery. In our patient, prolonged tube feeding, persistent vomiting, and severe dysphagia likely increased the risk of nutritional compromise, which may have further contributed to generalized deconditioning. Therefore, early nutritional assessment, appropriate nutritional support, and individualized swallowing rehabilitation should be considered complementary components of comprehensive rehabilitation to optimize functional recovery in patients with severe postoperative dysphagia [16].

### 3.4. Beyond Structural Correction: Functional Recovery and the Role of Comprehensive Rehabilitation

Most previous reports describing postoperative PMC and hydrocephalus have mainly focused on structural complications and local manifestations. Management strategies in these studies were primarily directed toward radiologic resolution and surgical correction of CSF-related abnormalities. However, little attention has been paid to functional impairments such as cognitive dysfunction and dysphagia or to functional recovery through rehabilitation.

In contrast, our case goes beyond structural abnormalities alone and highlights the substantial functional consequences associated with postoperative PMC and hydrocephalus. Severe cognitive impairment, dysphagia, and prolonged physical deconditioning led to marked functional deterioration. Importantly, comprehensive and individualized rehabilitation resulted in meaningful recovery of cognitive function, swallowing ability, and overall mobility. To our knowledge, reports focusing on rehabilitation-associated recovery following postoperative PMC and hydrocephalus after upper cervical fusion remain extremely limited. This case suggests that structural correction alone may be insufficient to optimize functional recovery and highlights the importance of integrating comprehensive multidisciplinary rehabilitation into the management of patients with rare postoperative neurological complications.

## 4. Conclusions

Postoperative CSF leakage-associated hydrocephalus and PMC may lead to severe cognitive, swallowing, and functional impairments beyond structural complications alone. Comprehensive, individualized rehabilitation should be considered an essential component of care to maximize neurological and functional recovery.

## Figures and Tables

**Figure 1:**
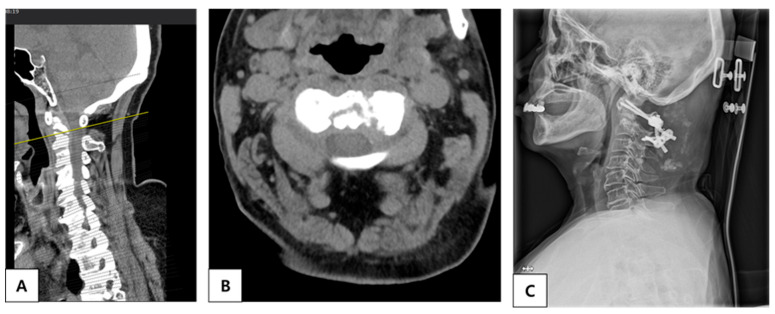
(**A**,**B**) Cervical spine CT images demonstrating a type III odontoid fracture involving the dens and extending into the C2 vertebral body with nonunion. (**C**) Postoperative cervical spine X-ray showing posterior C1–2 fusion.

**Figure 2:**
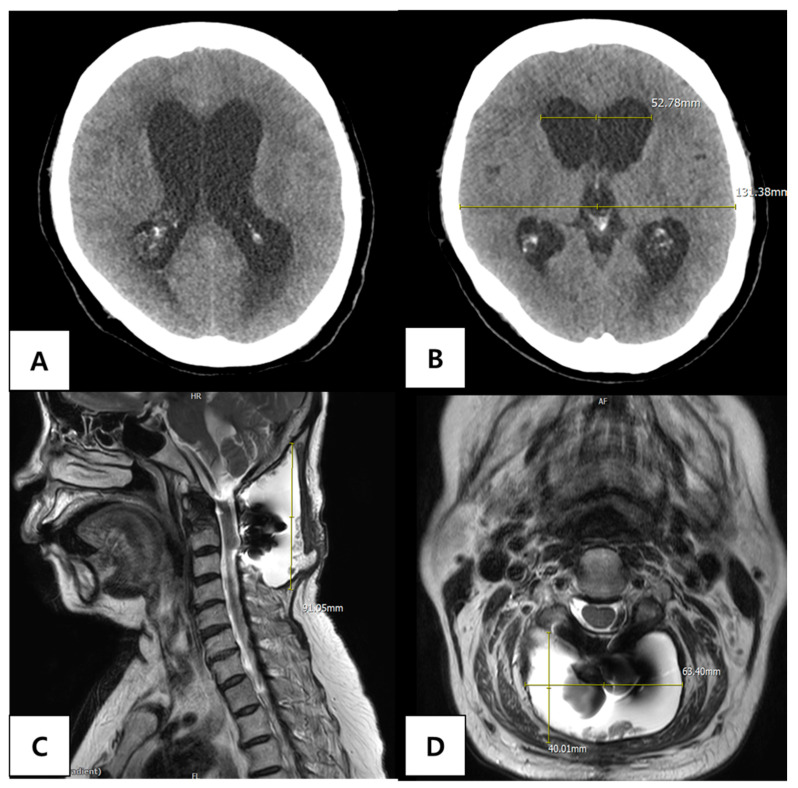
Postoperative hydrocephalus and pseudomeningocele secondary to cerebrospinal fluid leakage. (**A**,**B**) Axial brain CT demonstrating progressive ventricular enlargement consistent with hydrocephalus (Evans ratio = 0.40). (**C**,**D**) Cervical spine MRI demonstrating a giant postoperative pseudomeningocele associated with cerebrospinal fluid (CSF) leakage along the posterior surgical fixation site, measuring approximately 91 × 63 × 40 mm (length × width × depth).

**Figure 3:**
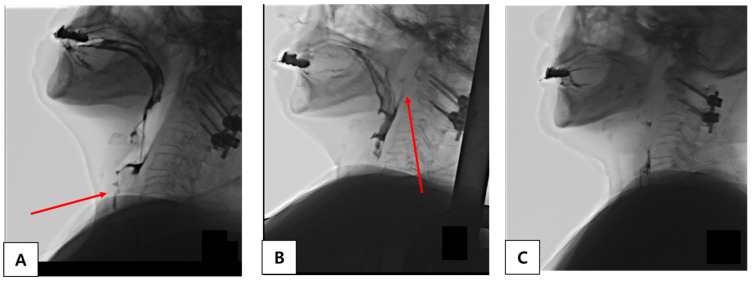
**Videofluoroscopic swallowing study findings during the clinical course.** (**A**) VFSS demonstrate severe silent aspiration (PAS 8), (**B**) Nasopharyngeal reflux observed during swallowing, (**C**). Red arrows indicate aspiration (**A**) and nasopharyngeal reflux (**B**). Follow-up VFSS demonstrate resolution of aspiration and improved swallowing function.

**Table 1:** Changes in Functional Evaluations throughout the Clinical Course.

	Postoperative 1 Month (Pre-Rehabilitation)	Postoperative 3 Month (Rehabilitation Initiation)	Postoperative 5 Month (After 2 Months of Rehabilitation)	Postoperative 6 Month (After 3 Months of Rehabilitation)
Cognitive function				
MMSE (0–30)	NA	5	9	19
Speech evaluation				
K-WAB (0–100)	NA	69.4	NA	85.5
Swallowing function				
FDS (0–100)	62	46	26	20
PAS (1–8)	8	8	5	1
DOSS	1	2	4	6
Oral Diet level (IDDSI 1–7)	NA	NA	4	7
Feeding method	Enteral feeding (L-tube)	Enteral feeding (L-tube)	Dysphagia diet	Regular diet
Motor & mobility function				
MFT (R/L,0–32)	22/21	27/23	29/26	32/26
MBI (0–100)	4	11	20	67
BBS (0–56)	3	3	5	39
FAC (0–6)	0	0	1	2
Mobility	Bed ridden	Sitting, wheel chair ambulation	Standing balance training	Walker ambulation

**Abbreviations:** NA, not assessed; MMSE, Mini-Mental State Examination; K-WAB, Korean version of the Western Aphasia Battery; FDS, Functional Dysphagia Scale; PAS, Penetration–Aspiration Scale; DOSS, Dysphagia Outcome and Severity Scale; IDDSI, International Dysphagia Diet Standardisation Initiative; MFT, Manual Function Test; MBI, Modified Barthel Index; BBS, Berg Balance Scale; FAC, Functional Ambulatory Category. Values in parentheses indicate the score range for each assessment. Higher FDS and PAS scores represent greater impairment; higher scores on all other measures represent better functional outcomes.

## Data Availability

The data supporting the findings of this case report are not publicly available due to patient confidentiality and privacy restrictions but may be available from the corresponding author upon reasonable request.

## Abbreviations

The following abbreviations are used in this manuscript:

ACDF	anterior cervical discectomy and fusion
BBS	Berg Balance Scale
CSF	cerebrospinal fluid
iNPH	idiopathic normal-pressure hydrocephalus
MMSE	Mini-Mental State Examination
MBI	Modified Barthel Index
PAS	Penetration-aspiration scale
PMC	Pseudomeningocele
VFSS	videofluoroscopic swallowing studies
